# Implicit Racial Bias and Unintentional Harm in Vascular Care

**DOI:** 10.1001/jamasurg.2024.7254

**Published:** 2025-02-26

**Authors:** Corey A. Kalbaugh, Erika T. Beidelman, Kerry A. Howard, Brian Witrick, Ashley Clark, Katharine L. McGinigle, Samantha Minc, Olamide Alabi, Caitlin W. Hicks, Andrew A. Gonzalez, Crystal W. Cené, Samuel Cykert

**Affiliations:** 1 Department of Epidemiology and Biostatistics, Indiana University School of Public Health–Bloomington, Bloomington; 2 Department of Public Health Sciences, Clemson University, Clemson, South Carolina; 3 Center for Public Health Modeling and Response, Clemson University, Clemson, South Carolina; 4 Center for Survey Research and O’Neill School of Public and Environmental Affairs, Indiana University–Bloomington, Bloomington; 5 Department of Surgery, School of Medicine, The University of North Carolina at Chapel Hill, Chapel Hill; 6 Department of Cardiovascular and Thoracic Surgery, West Virginia School of Medicine, Morgantown; 7 Department of Surgery, Emory University School of Medicine, Atlanta, Georgia; 8 Department of Surgery, Johns Hopkins University School of Medicine, Baltimore, Maryland; 9 Department of Surgery, Indiana University School of Medicine; Indianapolis; 10 Center for Health Services Research, Regenstrief Institute, Indianapolis, Indiana; 11 Department of Medicine, University of California at San Diego Health, La Jolla; 12 Division of General Medicine and Clinical Epidemiology, School of Medicine, The University of North Carolina at Chapel Hill, Chapel Hill

## Abstract

**Importance:**

Implicit bias may influence physician treatment decisions and contribute to Black-White health disparities. There are limited data linking implicit bias with actual care delivery and outcomes.

**Objective:**

To determine whether implicit racial bias is associated with potentially harmful surgical treatment selection for a cohort of patients with peripheral artery disease–related claudication.

**Design, Setting, and Participants:**

This survey study, linked with observational registry data, included eligible clinicians who participate in the Vascular Quality Initiative (VQI) among 960 centers. The VQI includes academic medical centers, teaching hospitals, community hospitals, and private practices. Eligible participants included all vascular specialist VQI members (N = 2512), of whom 218 completed the race implicit association test (IAT) and were linkable to procedure-level data. The study was conducted between October 2021 and October 2022.

**Exposure:**

Race IAT.

**Main Outcomes and Measures:**

Clinician-level implicit bias results were linked to patient-level registry data of peripheral revascularization procedures performed for claudication. The adjusted odds of performance of any infrapopliteal procedure by specialist implicit bias and patient race were measured via mixed-effects logistic regression models. Implicit bias as a moderator of the association of infrapopliteal procedures for claudication and patient race with 1-year amputation was assessed as a secondary outcome.

**Results:**

Among 218 vascular specialists (mean [SD] age, 46 [9] years; 160 [73%] male), 157 (72%) had a pro-White bias. Black patients treated by a physician with pro-White bias had a significant increase in the odds of receiving an infrapopliteal procedure compared with the total sample (adjusted odds ratio [AOR], 1.67; 95% CI, 1.12-2.48). When treated by a specialist with pro-White bias, Black patients had increased odds of 1-year amputation, regardless of anatomic location treated, compared with White patients (AOR, 2.34; 95% CI, 1.20-4.55). Conversely, Black patients treated by a specialist with no bias had similar odds of an infrapopliteal procedure (AOR, 0.93; 95% CI, 0.68-1.26) as the full patient sample and similar odds of 1-year amputation (AOR, 1.29; 95% CI, 0.33-4.99) as White patients.

**Conclusions and Relevance:**

These findings indicate that implicit bias is associated with potentially harmful infrapopliteal procedures for Black patients and contributes to Black-White outcome disparities in the US. These results suggest the need for system-level interventions that transparently identify and warn of procedures not aligned with best practices to reduce the negative influence of implicit bias.

## Key Points

**Question:**

Is physicians’ implicit racial bias associated with the provision of low-value care for Black or White patients with claudication?

**Findings:**

In this survey study, 72% of 218 vascular specialists had a pro-White implicit bias, and pro-White bias was associated with low-value care for Black patients.

**Meaning:**

These findings suggest that physicians who treat patients with vascular diseases must consider their own practices and where they may be falling short of standards, particularly for their Black patients.

## Teaser

This survey study examines whether physicians’ implicit racial bias is associated with potentially harmful surgical treatment selection for Black and White patients with peripheral artery disease–related claudication.

## Introduction

Nearly 10 million people in the US have claudication, a subtype of peripheral artery disease (PAD) associated with severe lifestyle limitations.^1^ Compared with White patients, Black patients with claudication have a higher risk of limb amputation despite claudication being a non–limb threatening condition.^2^ Our team has focused on claudication management and how differences in the delivery of health care to Black vs White patients are contributing to Black-White limb outcome inequities. We found that, medically, Black patients are less likely to be offered guideline-directed therapy, such as a preoperative statin^3^; and, surgically, Black patients with claudication are more likely to undergo early infrapopliteal peripheral revascularizations.^4,5^ These infrapopliteal interventions are not supported by current guidelines^6,7^ and expose patients to a higher risk of disease progression and major amputation than isolated femoropopliteal interventions or medical management alone.^8^

As a next step, our team has explored why Black patients receive different and suboptimal care compared with White patients even when controlling for location, insurance, socioeconomic status, and risk factor profiles. One potential mechanism contributing to this problem is implicit bias, defined as an unconscious bias that may influence decision-making.^9^ As described in several systematic reviews, health care professionals have implicit biases that may impact the delivery of health care in a variety of settings.^10,11,12^ For example, physician bias and stereotyping may have an influence on who receives the best health care interventions and who receives harmful care even if unintentional.^13,14^ There are limited data exploring the relationship between physicians’ implicit biases with actual care delivery and outcomes.

Here, we address this critical gap and present an approach to linking participant implicit bias results to those same participants’ delivery of care and associated outcomes. Specifically, we sought to examine whether physician implicit bias is associated with the provision of low-value care for Black or White patients with claudication using the implicit association test (IAT).^15^ We defined low-value care as the performance of infrapopliteal peripheral revascularization procedures for claudication. We also assessed the association between infrapopliteal procedures and patient race on the odds of a 1-year amputation, using physician implicit bias as a moderator.

## Methods

The institutional review boards at Clemson University and Indiana University approved this survey study, which was conducted between October 2021 and October 2022. Informed consent was obtained electronically as part of the survey. All data were administered and stored on a secure SSL encrypted website dedicated to this project. The website was managed by Project Implicit. A dataset may be provided to physicians who participate in the Society for Vascular Surgery’s Vascular Quality Initiative (VQI), pending approval of a research proposal. This study followed the American Association for Public Opinion Research (AAPOR) and the Strengthening the Reporting of Observational Studies in Epidemiology (STROBE) reporting guidelines.

### Study Sample and Source Population

The VQI is a vascular procedure–based registry^16^ intended to document and improve the quality of care delivered to patients with vascular diseases. Clinicians who participate in the delivery of vascular care in the US are eligible to participate in the VQI. As of January 2023, the VQI includes 960 sites. The VQI has 18 regional quality groups within 4 regions: South (5), Northeast (4), Midwest (4), and West (5). Centers include academic medical centers, teaching hospitals, community hospitals, and private practices. This study used data from the peripheral vascular intervention (PVI) and infrainguinal bypass registries of the VQI from 2012 to 2022.

We invited all vascular specialist VQI members (N = 2512) to take the race IAT. Participants self-reported their race and ethnicity as a field in the survey. We then linked each participant’s results to procedure and patient-level data from the VQI registry. The registry includes patient demographics, comorbid conditions, imaging studies, medication usage, periprocedural details, and in-hospital and 30-day outcomes. Patients also complete a 1-year follow-up visit for any procedure covered by the VQI, and outcome data at 1 year are included in the registry. The Social Security Death Index is linked to ascertain deaths. We restricted the procedure-level dataset to observations linked to White or Black patients because the race IAT only measures implicit bias in relation to White or Black individuals. Additionally, we restricted the sample to include only procedures performed in patients with claudication in the limb being intervened on. This restriction was chosen to isolate a risk > benefit procedure for examination, where, in patients with claudication, the procedure choice is not considered to be limb sparing.^6,7^

### Exposure: Implicit Bias

The IAT is an online assessment that takes 10 to 15 minutes to complete and asks participants to associate images of White and Black individuals with positive or negative attributes. The IAT calculates d-scores based on the differences in reaction times across sequential tests. These scores were then used to group participants into 7 implicit bias categories based on an observed racial preference: strong pro-White bias, moderate pro-White bias, slight pro-White bias, no bias, slight pro-Black bias, moderate pro-Black bias, and strong pro-Black bias. We then further reduced this categorical variable into 2 levels to compare physicians with any evidence of pro-White bias with physicians with no evidence of bias.

### Primary Outcome: Risk > Benefit Procedures (Infrapopliteal Interventions)

We defined infrapopliteal interventions as any intervention performed on the infrapopliteal segment with or without concomitant femoropopliteal involvement. The reference group for this exposure included only isolated femoropopliteal interventions. Because these infrapopliteal procedures often lead to major adverse vascular events, we used this as our primary outcome.^17^ Performance of an infrapopliteal procedure was represented by a binary indicator variable, with 1 indicating the procedure included the infrapopliteal segment and 0 indicating it did not.

### Secondary Outcome: 1-Year Amputation

The VQI registry longitudinally follows patient outcomes for each procedure, including major amputation. Major amputation included all above or below the knee with loss of the entire foot. Therefore, we examined rates of major amputation within 1 year of the recorded procedure. Individuals with less than 1 year of follow-up and no recorded amputation event were excluded from the analysis.

The use of VQI follow-up data is limited by some loss to follow-up prior to 1-year. In our subsample, approximately 12% of procedures were missing follow-up data. Rates of loss to follow-up prior to 1-year were largely nondifferential across procedures performed by physicians with pro-White bias (11%) and no bias (13%). Sensitivity analyses were employed to determine the potential impact of this missingness.

### Covariates

Physician, procedure, and patient-level covariates were included in our analysis to describe the sample and account for confounding (Table 1 and Table 2). Physician-level characteristics included age, sex, specialty, years of experience, race and ethnicity, and region. Procedure-level characteristics included year of procedure, number of vascular beds, anatomic location of the intervention, and type of intervention. Patient-level characteristics included age, sex, race and ethnicity, insurance status, diabetes status, dialysis status, current smoking status, body mass index, and disease severity. Procedure year was treated as a factor variable within all analyses.

**Table 1.** Physician Characteristics Across Implicit Bias Categories

Characteristic	Pro-Black bias (n = 23)	No bias (n = 38)	Pro-White bias (n = 157)	*P* value^a^
Age, mean (SD), y	47 (11)	44 (7)	46 (10)	.73
Sex, No. (%)^b^				
Female	8 (36)	7 (18)	41 (26)	.31
Male	14 (64)	31 (82)	115 (74)	
Procedures, median (IQR)	12.5 (6-35)	14.5 (4-50)	16 (6-35)	.79
Specialty, No. (%)				
Cardiothoracic surgery	0	0	1 (1)	.92
Interventional cardiology	1 (4)	2 (5)	7 (4)	
Interventional radiology	1 (4)	1 (3)	7 (4)	
Vascular surgery	21 (91)	35 (92)	142 (90)	
Years of experience, No. (%)				
0-2	2 (9)	0	19 (12)	NR
3-5	6 (26)	8 (21)	24 (15)	
6-10	4 (17)	18 (47)	44 (28)	
11-15	2 (9)	6 (16)	23 (15)	
16-20	2 (9)	3 (8)	10 (6)	
21-25	3 (13)	0	18 (11)	
≥26	4 (17)	3 (8)	19 (12)	
Race and ethnicity, No. (%)				
Hispanic	2 (9)	1 (3)	6 (4)	.046^a^
Non-Hispanic Black	3 (13)	3 (8)	4 (3)	
Non-Hispanic White	14 (61)	20 (53)	110 (70)	
Non-Hispanic other^c^	4 (17)	14 (37)	37 (24)	
Region, No. (%)				
Midwest	2 (9)	9 (24)	34 (22)	.68
Northeast	6 (26)	7 (18)	41 (26)	
South	11 (48)	18 (47)	61 (39)	
West	4 (17)	4 (11)	21 (13)	

Abbreviation: NR, not reported.

^a^ Fisher exact test; Mood’s Median test; Kruskal-Wallis rank sum test; Pearson χ^2^ test.

^b^ Data on sex were missing for 1 physician in the pro-Black bias group and 1 physician in the pro-White bias group.

^c^ Other race and ethnicity includes American Indian or Alaska Native, Asian, Native Hawaiian or Other Pacific Islander, more than 1 race, or unknown.

**Table 2.** Patient Characteristics Overall and by Patient Race

Characteristic	No. (%) of patients			*P* value^a^
	Overall (N = 6588)	White (n = 5399)	Black (n = 1189)	
Age, mean (SD), y	67 (10)	67 (10)	65 (10)	<.001
BMI, mean (SD)	28.4 (6.0)	28.4 (5.9)	28.3 (6.4)	.36
Male	4335 (66)	3687 (68)	648 (54)	<.001
Current smoker	2635 (40)	2110 (39)	525 (44)	.001
Diabetes	2826 (43)	2222 (41)	604 (51)	<.001
Severe disease^b^	2671 (40)	2160 (40)	511 (43)	.24
Has insurance	5641 (86)	4660 (86)	981 (82)	.005
Has commercial insurance	2247 (34)	1890 (35)	357 (30)	.001
Bypass	1641 (25)	1386 (26)	255 (21)	.003
>1 Vascular bed	1188 (18)	979 (18)	209 (18)	.19
Infrapopliteal^c^	880 (14)	661 (13)	219 (19)	<.001

Abbreviation: BMI, body mass index (calculated as weight in kilograms divided by height in meters squared).

^a^ Wilcoxon rank sum test; Pearson χ^2^ test.

^b^ For procedures occurring after September 22, 2016, disease severity was classified directly via the Vascular Quality Initiative leg symptom and indication variables. For procedures before this date, we used patient ankle brachial index (ABI) to classify disease severity. An ABI of 0.70 to 1.40 was considered mild, 0.40 to 0.69 was moderate, and 0 to 0.39 was severe. An ABI above 1.40 was considered noncompressible and categorized as severe disease. When ABI was not present, we used toe-brachial index (TBI) or toe pressure to classify disease severity where available (TBI ≥0.70 = mild; TBI 0.40-0.69 = moderate; TBI <0.40 = severe).

^c^ Infrapopliteal is defined as any intervention performed on the infrapopliteal segment with or without concomitant femoropopliteal involvement.

### Statistical Analysis

To reduce the potential influence of differences in the characteristics of physicians from the Society for Vascular Surgery VQI registry who participated in our study compared with the full registry, data were weighted according to widely used practices to adjust for nonresponse bias (eMethods and eTable 1 in Supplement 1).^18,19^

We conducted univariate analyses to describe the characteristics of physicians and procedures in our sample, comparing physician demographics across the 3 implicit bias groups. To address our research questions, we focused on procedures for patients with claudication and used mixed-effects logistic regression models to examine the association between physician implicit bias and infrapopliteal procedures. The models estimated the odds of performing this procedure by bias category, adjusting for variables such as physician race, procedure year, patient demographics, and disease factors. Each model included a random intercept for the physician to account for correlations in procedures performed by the same doctor, with physicians grouped by VQI centers to account for similarities within each center. We also included an interaction term between bias category and patient race to explore whether bias influenced procedure choices differently for Black vs White patients. Interaction contrasts showed the odds of infrapopliteal procedures for each subgroup compared with the overall sample.

To examine the association between infrapopliteal procedures, patient race, and 1-year amputation risk, we used implicit bias as a moderating factor in additional mixed-effects models. We stratified analyses by physician bias levels but could not include a pro-Black bias category due to limited data. A sensitivity analysis treated missing follow-up data as indicating no amputation to gauge how these cases might affect results.

## Results

### Sample Vascular Specialists Characteristics

There were 218 vascular specialists who met the inclusion criteria of (1) completing the entire IAT survey and (2) being able to be linked to procedure-level data in the VQI. A total of 198 physician participants were vascular surgeons (91%); 9 were Hispanic (4%), 10 were non-Hispanic Black (5%), 144 were non-Hispanic White (66%), and 55 were other race or ethnicity (25%); and 56 were female (26%) and 160 male (73%) (2 were missing data on sex). Participants’ mean (SD) age was 46 (9) years (Table 1).

### Sample Procedure and Patient Characteristics

These 218 vascular specialists were linkable to 22 590 revascularization procedures (median [IQR], 15 [6-37] procedures). Specialists performed a median (IQR) of 15 (6-33) endovascular interventions and 6 (2-15) bypass interventions. A peripheral revascularization procedure for claudication was performed in 6588 patients. Most of these procedures were endovascular (4947 [75%]), and 1188 (18%) included more than 1 vascular bed (Table 2). Compared with Black patients, White patients had a higher likelihood of bypass procedures (1386 [26%] vs 255 [21%]; *P* = .003), but no differences were observed in the number of treated vascular beds by race. Additionally, a higher proportion of Black patients were treated with an infrapopliteal procedure compared with White patients (219 [19%] vs 661 [13%]; *P* < .001). Compared with White patients, Black patients were younger, more likely to be female, and had a higher prevalence of current smoking status and diabetes (Table 2).

There were 880 infrapopliteal procedures, including 275 infrapopliteal bypasses, 160 isolated tibial PVI procedures, and 321 infrapopliteal PVI interventions with concomitant femoropopliteal involvement. Patients undergoing infrapopliteal procedures were more likely to be male (627 [71%] vs 3512 [65%]), be a nonsmoker (267 [30%] vs 2218 [41%]), have diabetes, and be of Black race (*P* < .001 for all) (eTable 2 in Supplement 1). Specialists performed a median (IQR) of 3 (1-7) infrapopliteal interventions. Twenty-seven specialists performed 10 or more infrapopliteal interventions (maximum of 42).

### Physician Implicit Bias

Based on the results of the IAT, we found that 72% of vascular specialists had a pro-White bias (n = 157), 17% had no specific preference for Black or White individuals (n = 38), and 11% had a pro-Black bias (n = 23). Among 157 specialists with a pro-White bias, 39 (25%) demonstrated slight bias, 70 (45%) moderate bias, and 48 (31%) strong bias. Of the 23 specialists with a pro-Black bias, 10 (43%) demonstrated slight, 10 (43%) moderate, and 3 (13%) strong bias.

### Physician Implicit Bias and Infrapopliteal Procedures

From an adjusted model, we found a significant interaction between physician pro-White bias and patient race on the odds of an infrapopliteal procedure among patients with claudication (adjusted odds ratio [AOR], 1.62; 95% CI, 1.02-2.57) (Table 3). Black patients treated by a physician with pro-White bias had a significant increase in the odds of receiving an infrapopliteal procedure compared with the total sample (AOR, 1.67; 95% CI, 1.12-2.48) (Figure). In contrast, White patients treated by physicians with pro-White bias or no bias had reduced odds of an infrapopliteal procedure compared with the sample (physician with pro-White bias: AOR, 0.84; 95% CI, 0.70-1.01; physician with no bias: AOR, 0.76; 95% CI, 0.63-0.90). The effect estimates for all model covariates are available in eTable 3 in Supplement 1. We also found no significant differences in the odds of an intervention involving more than 1 vascular bed across physician implicit bias categories and patient race (eTable 4 in the Supplement).

**Table 3.** Odds of an Infrapopliteal Procedure Among Patients With Claudication

Variable	Unadjusted		Adjusted^a^	
	Odds ratios (95% CI)	*P* value	Odds ratios (95% CI)	*P* value
Bias				
None	1 [Reference]	NA	1 [Reference]	NA
Pro-White	1.12 (0.78-1.60)	.54	1.10 (0.76-1.60)	.61
Patient race				
White	1 [Reference]	NA	1 [Reference]	NA
Black	1.30 (0.88-1.91)	.18	1.24 (0.82-1.88)	.31
Bias × race interaction				
No bias × Black patient race	1 [Reference]	NA	1 [Reference]	NA
Pro-White bias × Black patient race	1.42 (0.91-2.19)	.12	1.62 (1.02-2.57)	.04

Abbreviation: NA, not applicable.

^a^ Adjusted for physician race, year, patient sex, patient smoking status, patient diabetes status, patient age, patient dialysis status, and patient disease severity. Random intercept accounts for physician correlation nested within Vascular Quality Initiative centers.

**Figure.**
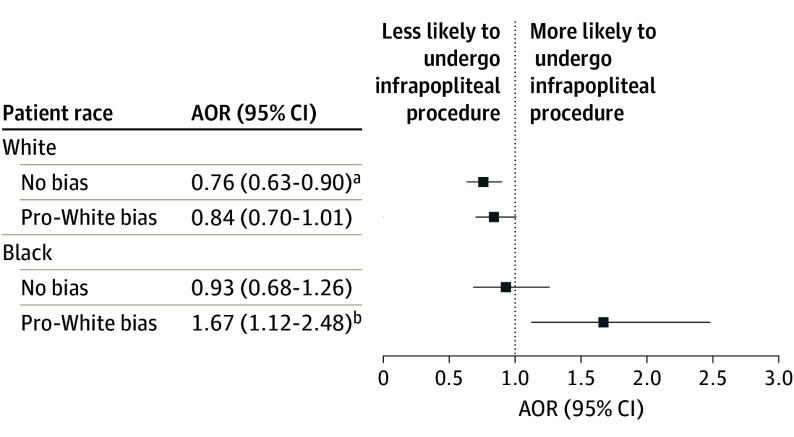
Interaction Contrasts From the Adjusted Infrapopliteal Procedure Model AOR indicates adjusted odds ratio. ^a^*P* = .04. ^b^*P* < .001.

### 1-Year Incidence of Major Amputation

Crude 1-year incidence and incidence rates are shown in eTable 5 in Supplement 1. The overall 1-year incidence rate of major amputation was 1.10 (95% CI, 0.77-1.30) per 100 person-years in the population of patients treated for claudication. Incidence of amputation was higher among Black compared with White patients (1.94% vs 0.81%; adjusted hazard ratio, 2.14; 95% CI, 1.23-3.72).

### 1-Year Amputation and Physician Implicit Bias as a Moderator

Among patients treated by a physician with no racial bias, patient race was not associated with the odds of 1-year amputation (AOR, 1.29; 95% CI, 0.33-4.99) (Table 4). In contrast, among patients treated by physicians with a pro-White bias, Black patients had increased odds of experiencing an amputation within 1 year of the procedure compared with White patients (AOR, 2.34; 95% CI, 1.20-4.55). In the sensitivity analysis (eTable 6 in Supplement 1) in which all individuals missing 1-year follow-up data were coded as experiencing no amputation, the magnitude of our results changed slightly, but the direction and significance of the results was retained.

**Table 4.** Odds of 1-Year Amputation Associated With Patient Race Stratified by Implicit Bias Category, Among Patients With Claudication

Patient race	No bias		Pro-White bias	
	Odds ratios (95% CI)^a^	*P* value	Odds ratios (95% CI)^a^	*P* value
White	1 [Reference]	NA	1 [Reference]	NA
Black	1.29 (0.33-4.99)	.71	2.34 (1.20-4.55)	.01

Abbreviation: NA, not applicable.

^a^ Adjusted for physician race, year, patient gender, patient smoking status, patient diabetes status, patient age, patient dialysis status, and patient disease severity. Random intercept accounts for physician correlation nested within Vascular Quality Initiative centers.

## Discussion

The influence of implicit bias on clinical decision-making has been examined as a potential contributor to health inequities. However, most prior work has used indirect comparisons of patient-clinician communication^20,21^ and mock clinical vignettes.^22,23^ To our knowledge, this study is the first to link formal implicit bias evaluations with actual care delivery and clinical outcomes. We chose the use of invasive infrapopliteal interventions as our measure of care and, for both White and Black patients, found these interventions were more prevalent than current guidelines support.^6,7^ An understanding of excess interventions is important because natural history studies suggest that three-fourths of people with claudication can be medically treated without experiencing significant symptom progression in their lifetime.^24^ The risk of major lower extremity amputation for people with claudication is 1% to 2% over 5 years with optimized medical treatment.^6^ Still, the US has seen a rapid increase in the surgical management of claudication, with more interventions to infrapopliteal arteries.^25^ Patients undergoing bypass surgery to infrapopliteal arteries have more than double the risk of amputation compared with patients with an above-knee procedure.^6^ Patients undergoing infrapopliteal PVI have at least a 2-fold greater risk of limb loss at 1 year compared with natural history estimates.^7^ Such low-value care can become limb-risking care.

Past reports suggest the majority of the US population has some form of implicit bias. It is neither concerning nor surprising that the IAT results among these vascular specialists parallel those of the general population and other health care fields^10,11,12^; however, it is concerning that the output is associated with greater use of likely harmful interventions ultimately associated with a documented increase in limb amputation. Our moderation analysis provides early evidence that these higher rates of amputation documented among Black patients with PAD are partially explained via implicit bias and its impact on treatment decisions for Black patients. Notably, we found that physician implicit bias compounds with the negative outcomes of infrapopliteal procedures to put Black patients at significantly higher risk of 1-year amputations. Though infrapopliteal procedures for revascularization are associated with higher amputation rates among both Black and White patients, Black patients with a physician with pro-White bias are far more likely to have this procedure type selected. Fewer than 1 in 5 specialists had a no bias result. Importantly, specialists with no demonstrated bias were less likely to perform a low-value procedure on either their Black or White patients and had the least treatment variability between Black and White patients.

To lessen these disparities, we advocate for a multilevel intervention similar to the ACCURE study, which mitigated Black-White disparities in completion of treatment and outcomes for early-stage breast and lung cancer.^26,27^ The first component was real-time transparency through an electronic health record–derived registry and warning system that alerted clinical teams not only to missed patient appointments but also missed expected milestones in care, with the latter process identifying clinical inertia. The second component was an accountability system in which clinical teams were presented their quarterly data on treatment completion according to race and would brainstorm on reversible barriers leading to these differences. The third component was enhanced communication through a navigator who would ensure timely and clear communication with patients and advocacy for the patient with the clinical team. Compared with both historical and concurrent controls, this system-based intervention not only reduced substantial Black-White gaps in cancer care completion but also significantly increased care completion for White patients.^26^ Similar informatics support, audit and feedback, and enhanced communication could lead to optimized care for vascular patients that maximizes medical care and highlights appropriate procedure use. The VQI has already championed and successfully completed numerous national-level quality improvement projects, including significantly increasing the prescription of class Ia recommended antiplatelets and statins among people undergoing a peripheral revascularization procedure. In a separate study, we found that implicit bias was not associated with the delivery of those guideline-directed (and monitored) medications.^28^ Similar resources could be leveraged to reduce the use of infrapopliteal revascularization for claudication, which is not as closely governed and is affected by implicit bias. Our team also advocates for the vital role of implementation science in understanding how to optimize care delivery. We point to HDPulse^29^ as a robust online repository that includes ACCURE and similar interventions that have been successful in reducing health disparities.

Notably, the IAT measures implicit bias^10^ and not any overt racism or malintent. Still, whatever the processes are that lead to care differences must be mitigated. The stakes are high when physicians who make limb and life decisions for people have biases that may adversely affect people’s lives. Unfortunately, to date, interventions aimed at reducing individual-level implicit bias have not had demonstrated sustained impact. Instead, to take the necessary step of mitigating these inequities, experts in implicit bias describe the importance of disparity finding in large organizations.^30^ We suggest that insurance providers and hospital systems should routinely be reviewing their organization-level data to identify places where disparate care is administered and where associated outcomes are worse for historically disadvantaged groups, and build system-based interventions analogous to ACCURE. To further encourage change, individual and organizational incentives could be provided to reward high-value care on a population and individual patient level. Such payments for value-based care could include bonuses for achieving care excellence and equity instead of the fee-for-service model that may encourage utilization of procedures such as the infrapopliteal interventions for claudication described in our study.

### Strengths and Limitations

Our study has a number of strengths. This is, to our knowledge, the largest survey of implicit bias among vascular specialists and the only study to link the implicit bias score of a specialist to a registry with actual delivered clinical care and resulting outcomes. Thus, our study represents a major step forward in understanding the important role that unconscious bias plays in the delivery of low-value care to vascular patients. Instead of documenting disparities, our study indicates the role of unconscious bias as a potential actionable mechanism behind vascular disparities.

This study also has some limitations. There is an ongoing debate over the use of the IAT to measure implicit bias.^31,32^ Due to practical constraints on time, interest, and funding, each participant in this study took the IAT only once, and results might vary if retested. Still, the IAT has a test-retest reliability score of r = 0.50, and most people get similar results over multiple tests. The IAT is not perfect, but it is widely used for assessing unconscious bias.^30,33,34^ Given sample size issues, we were not able to examine the severity of bias (slight, moderate, strong). Regardless, the IAT remains the most widely used test for studying unconscious bias with good reliability and validity.^35^ A limitation of our data source (VQI) is that it lacks information on why revascularization was chosen over continued medical management. For example, we do not have information on whether an intervention was performed to maintain employment and to correct truly lifestyle-limiting claudication. Still, it is important to note that, even if done with good intentions, these interventions are often harmful and have unintended consequences. We only included vascular specialists who participate in the lower-extremity modules of the VQI. Further, our sample included more women, younger specialists, and fewer from the Midwest than the overall frame. Because we anticipated the aforementioned challenges, we were able to make inferences to the broader VQI vascular specialist population.

## Conclusions

In this study, we provide evidence that implicit bias is present for many vascular clinicians who treat PAD, and this implicit bias was associated with increased delivery of low-value, potentially harmful care for Black patients. This bias, while unintentional, was further associated with poor outcomes when paired with low-value care. These findings indicate a need for system-level interventions to identify and warn of procedures not aligned with best practices as a way to reduce the negative influence of implicit bias.

## Notes

Supplement 1.
eMethods.
**eTable 1.** Demographic Characteristics of the Full Physician Frame and Sample - Implicit Bias Study **eTable 2.** Patient Characteristics by Procedure Type (Non-Infrapopliteal vs. Infrapopliteal) **eTable 3.** Effect Estimates for Covariates Included in Table 2 **eTable 4.** Odds of a Procedure Including >1 Vascular Beds Among Patients with Claudication **eTable 5.** One-Year Incidence Rate and One-Year Cumulative Incidence of Amputation by Physician Bias and Patient Race **eTable 6.** Sensitivity Analysis of Odds of 1-Year Amputation Associated with Patient Race Stratified by Implicit Bias Category, among patients with claudication

Supplement 2.
Data Sharing Statement
