# Supplementary material for: Implicit Racial Bias and Unintentional Harm in Vascular Care
Source: JAMA Surg. 2025 Feb 26;160(5):536–43. doi: 10.1001/jamasurg.2024.7254 (PMC11866068; doi:10.1001/jamasurg.2024.7254)
Supplement: Supplement 1. — eMethods. eTable 1. Demographic Characteristics of the Full Physician Frame and Sample - Implicit Bias Study eTable 2. Patient Characteristics by Procedure Type (Non-Infrapopliteal vs. Infrapopliteal) eTable 3. Effect Estimates for Covariates Included in Table 2 eTable 4. Odds of a Procedure Including >1 Vascular Beds Among Patients with Claudication eTable 5. One-Year Incidence Rate and One-Year Cumulative Incidence of Amputation by Physician Bias and Patient Race eTable 6. Sensitivity Analysis of Odds of 1-Year Amputation Associated with Patient Race Stratified by Implicit Bias Category, among patients with claudication [file jamasurg-e247254-s001.pdf]

Supplemental Online Content

Kalbaugh CA, Beidelman ET, Howard KA, et al. Implicit racial bias and unintentional harm in vascular care. *JAMA Surg*. Published online February 26, 2025.
doi:10.1001/jamasurg.2024.7254

eMethods.
eTable 1. Demographic Characteristics of the Full Physician Frame and Sample - Implicit Bias Study
eTable 2. Patient Characteristics by Procedure Type (Non-Infrapopliteal vs. Infrapopliteal)
eTable 3. Effect Estimates for Covariates Included in Table 2
eTable 4. Odds of a Procedure Including >1 Vascular Beds Among Patients with Claudication
eTable 5. One-Year Incidence Rate and One-Year Cumulative Incidence of Amputation by Physician Bias and Patient Race
eTable 6. Sensitivity Analysis of Odds of 1-Year Amputation Associated with Patient Race Stratified by Implicit Bias Category, among patients with claudication

This supplemental material has been provided by the authors to give readers additional information about their work.

© 2025 Kalbaugh CA et al. JAMA Surgery.

eMethods

Implicit Association Test. The IAT is the most widely used test for studying unconscious bias in scientific research with established acceptable levels of reliability and validity.[1,2] The average test-retest reliability of the IAT has been measured as r = 0.50, where a majority of individuals who take the test multiple times remain on the same side of zero as their first test.[3-5] To further ensure the validity of results we assessed differences in implicit bias findings by the self-reported number of times the participant has taken the IAT and the device used (computer, phone).

Weighting. First, we cross-classified physicians into categories (or "weighting cells") based on the following three variables: US geographic region, age category, and sex, due to differences in the likelihood of participating in the study (for example, physicians who identified as female were more likely to participate). Then, a weight defined as the number of physicians on the registry divided by the number of physicians who participated in the study was applied within each "weighting cell" (e.g., male physicians 30-44 years of age from the Northeast region of the United States) to "weight up" physicians from demographic groups who were underrepresented in the data. Analyses presented in our manuscript account for weighting.

Statistical Analysis. We then performed univariate analyses to characterize the physicians and procedures included in our sample. We compared the distribution of physician-level sociodemographic characteristics across the three identified implicit bias categories. To answer our research questions, we restricted our dataset to procedures linked to patients that presented with claudication. To assess the association between physician implicit bias and the performance of infrapopliteal interventions among patients with claudication, we fit mixed effects logistic regression models. With these models we estimated the odds of an infrapopliteal procedure being performed associated within each implicit bias category. Models were adjusted for physician race, procedure year, patient gender, smoking status, diabetes status, age, dialysis status, and disease severity. All models included a random intercept for the physician to account for correlation between multiple procedures performed by the same physician. Within the random intercept, physicians were nested within the VQI center that the procedure was performed to account for additional correlation between physicians at each VQI center. Additionally, all models included an interaction between implicit bias category and patient race. We included this interaction term as we anticipated that the association between implicit bias and procedure performance would vary based on patient race/ethnicity. We also report interaction contrasts for the adjusted model which compare the odds of an infrapopliteal procedure associated with each interaction sub-group to the sample average.

We assessed the association between both infrapopliteal procedures and patient race on the odds of a 1-year amputation, using physician implicit bias as a moderator. We estimated all associations via mixed effects models with a random intercept for the physician to account for the correlation between procedures performed by the same physician. To assess moderation by physician implicit bias, we stratified our analysis by physician implicit bias. Due to insufficient sample size, we could not fit a model for the pro-Black bias category in the stratified models assessing the associations with amputation. Additionally, we performed a sensitivity analysis for these models to assess the potential impact of under-representation of individuals experiencing no post-operative complications. For this, we categorized all individuals missing follow-up data as experiencing no amputation event to simulate the most extreme case of not at random missingness for this outcome. All analyses were performed in RStudio version 4.1.1 and the mixed effects models were fit using the lme4 package.

1. Greenwald A, McGhee D, Schwarz J. Measuring individual differences in implicit cognition: the implicit association test. . *J Pers Soc Psychol*. 1998;74:1464–1480.
2. Greenwald AG, Poehlman TA, Uhlmann EL, Banaji MR. Understanding and using the Implicit Association Test: III. Meta-analysis of predictive validity. *J Pers Soc Psychol*. 2009;97:17-41. doi: 10.1037/a0015575
3. Greenwald AG, Dasgupta N, Dovidio JF, Kang J, Moss-Racusin CA, Teachman BA. Implicit-Bias Remedies: Treating Discriminatory Bias as a Public-Health Problem. *Psychol Sci Public Interest*. 2022;23:7-40. doi: 10.1177/15291006211070781
4. Nosek BA, Smyth FL, Hansen JJ, Devos T, Lindner NM, Ranganath KA, Smith CT, Olson KR, Chugh D, Greenwald AG, et al. Pervasiveness and correlates of implicit attitudes and stereotypes. *European Review of Social Psychology*. 2007;18:36-88. doi: 10.1080/10463280701489053
5. Greenwald AG, Lai CK. Implicit Social Cognition. *Annu Rev Psychol*. 2020;71:419-445. doi: 10.1146/annurev-psych-010419-050837

© 2025 Kalbaugh CA et al. JAMA Surgery.

eTable 1. Demographic Characteristics of the Full Physician Frame and Sample - Implicit Bias Study

	Full Frame	Sample (unweighted)	Sample (weighted)
**Region**			
Midwest	29.3	20.6	28.8
Northeast	21.3	24.8	21.8
South	36.3	41.3	35.6
West	13.2	13.3	13.8
**Race/Ethnicity**			
Hispanic	3.7	4.1	2.8
Non-Hispanic Black	3.1	4.6	3.3
Non-Hispanic White	68.0	66.1	68.8
Non-Hispanic Other	25.2	25.2	25.1
**Sex**			
Male	89.5	74.3	85.9
Female	10.5	25.7	14.1
**Age**			
30-44	29.8	57.3	29.8
45-54	33.8	23.9	38.9
55+	36.5	18.8	31.3
**Academic**			
Yes	72.3	71.6	71.6
No	27.7	28.4	28.4
	N=2,512	n=218	n=218

© 2025 Kalbaugh CA et al. JAMA Surgery.

eTable 2. Patient Characteristics by Procedure Type (Non-Infrapopliteal vs. Infrapopliteal)

**Characteristic**	**Non-Infrapopliteal** N = 5,401[1]	**Infrapopliteal** N = 880[1]	**p-value**[2]
Age	67 (10)	67 (11)	0.1
BMI	28.4 (6.0)	28.6 (5.9)	0.4
Male	3,512 (65%)	627 (71%)	**<0.001**
Current Smoker	2,218 (41%)	267 (30%)	**<0.001**
Diabetes	2,277 (42%)	440 (50%)	**<0.001**
Severe Disease	2,324 (43%)	346 (39%)	0.2
Has Insurance	4,627 (86%)	751 (85%)	0.9
Has Commercial Insurance	1,874 (35%)	278 (32%)	0.07
Bypass	1,059 (20%)	275 (31%)	**<0.001**
>1 Vascular Bed	743 (14%)	445 (50%)	**<0.001**
Black Race	921 (17%)	219 (25%)	**<0.001**

[1] Infrapopliteal defined as any intervention performed on the infrapopliteal segment with or without concomitant femoropopliteal involvement

© 2025 Kalbaugh CA et al. JAMA Surgery.

eTable 3: Effect Estimates for Covariates Included in Table 2

Effect	Odds ratio	95% Confidence interval	p-value
**Year – 2012**	1.86	0.92 – 3.73	0.082
**Year – 2013**	1.35	0.67 – 2.71	0.405
**Year – 2014**	1.29	0.65 – 2.55	0.461
**Year – 2015**	1.29	0.66 – 2.52	0.460
**Year – 2016**	1.29	0.66 – 2.52	0.455
**Year – 2017**	1.54	0.80 – 2.95	0.194
**Year – 2018**	1.76	0.92 – 3.38	0.088
**Year – 2019**	1.11	0.58 – 2.15	0.747
**Year – 2020**	1.19	0.62 – 2.32	0.600
**Year – 2021**	1.06	0.55 – 2.06	0.854
**Year – 2022**	1.38	0.68 – 2.80	0.365
**Physician Race: Non-Hispanic White**	1.12	0.82 – 1.52	0.476
**Patient Gender: Male**	0.62	0.53 – 0.73	**<0.001**
**Patient Smoking: Current**	0.66	0.56 – 0.78	**<0.001**
**Patient Age (years)**	1.01	1.00 – 1.02	**0.014**
**Patient on Dialysis: Yes**	1.75	1.19 – 2.58	**0.005**
**Disease Severity: Moderate**	0.82	0.67 – 1.01	0.061
**Disease Severity: Severe**	0.77	0.61 – 0.96	**0.018**
**Patient Diabetic: Yes**	1.29	1.11 – 1.49	**0.001**

Reference Groups: Physician Race = Not Non-Hispanic White; Patient Gender = Female; Patient Smoking = Never or Ever Smoker; Patient on Dialysis = No; Disease Severity = Mild; Patient Diabetic = No.

© 2025 Kalbaugh CA et al. JAMA Surgery.

eTable 4. Odds of a Procedure Including >1 Vascular Beds Among Patients with Claudication

	Adjusted		
Predictors	*Odds Ratios*	*CI*	*p*
No Bias		*Reference*	
Pro-White Bias	1.24	0.88 – 1.74	0.2
White Patient Race		*Reference*	
Black Patient Race	0.72	0.47 – 1.10	0.1
No Bias*Black Patient Race		*Reference*	
Pro-White Bias*Black Patient Race	1.28	0.80 – 2.05	0.3

Adjusted for physician race, year, patient gender, patient smoking status, diabetes status, patient age, patient dialysis status, and patient disease severity. Random intercept accounts for physician correlation nested within VQI centers.

© 2025 Kalbaugh CA et al. JAMA Surgery.

eTable 5. One-Year Incidence Rate and One-Year Cumulative Incidence of Amputation by Physician Bias and Patient Race

	1-Year Incidence Rate*	1-Year Cumulative Incidence
Overall	1.10 (0.77, 1.30)	1.07% (62)
Pro-White Bias, White Patients	0.69 (0.44, 1.04)	0.74% (23)
Pro-White Bias, Black Patients	2.21 (1.29, 3.54)	2.38% (17)
No Bias, White Patients	1.04 (0.57, 1.74)	1.08% (14)
No Bias, Black Patients	0.98 (0.12, 3.52)	1.04% (2)

*Per 100 person-years

© 2025 Kalbaugh CA et al. JAMA Surgery.

eTable 6. Sensitivity Analysis of Odds of 1-Year Amputation Associated with Patient Race Stratified by Implicit Bias Category, among patients with claudication

	No Bias			Pro-White Bias		
Predictors	*Odds Ratios*	*CI*	*p*	*Odds Ratios*	*CI*	*p*
White Patient Race		*Reference*			*Reference*	
Black Patient Race	1.23	0.32 – 4.88	0.765	**2.24**	**1.15 – 4.36**	**0.018**

Sensitivity analysis classified all individuals with missing follow-up data as experiencing no amputation within 1-year of their procedure date. Adjusted for physician race, year, patient gender, patient smoking status, diabetes status, patient age, patient dialysis status, and patient disease severity.

© 2025 Kalbaugh CA et al. JAMA Surgery.
