# Supplementary material for: Implicit Racial Bias and Unintentional Harm in Vascular Care
Source: JAMA Surg. 2025 Feb 26;160(5):536–43. doi: 10.1001/jamasurg.2024.7254 (PMC11866068; doi:10.1001/jamasurg.2024.7254)
Supplement: Supplement 2. — Data Sharing Statement [file jamasurg-e247254-s002.pdf]

Data Sharing Statement

Kalbaugh. Implicit Racial Bias And Unintentional Harm In Vascular Care. *JAMA Surg*. Published February 26, 2025. doi:10.1001/jamasurg.2024.7254

Data
Data available: Yes
Data types: Deidentified participant data, Data dictionary
How to access data: ckalbaug@iu.edu
When available: With publication

Supporting Documents
Document types: None

Additional Information
Who can access the data: Researchers whose proposed use of the data has been approved
Types of analyses: For any purpose
Mechanisms of data availability: Signed data access agreement
Any additional restrictions: According to the bylaws of the SVS, the data from this study are available only for those who participate in the Vascular Quality Initiatve.
